# Understanding the Biosynthetic Changes that Give Origin to the Distinctive Flavor of Sotol: Microbial Identification and Analysis of the Volatile Metabolites Profiles During Sotol (*Dasylirion* sp.) Must Fermentation

**DOI:** 10.3390/biom10071063

**Published:** 2020-07-16

**Authors:** Francisco Javier Zavala-Díaz de la Serna, Ricardo Contreras-López, L. Paola Lerma-Torres, Francisco Ruiz-Terán, Beatriz A. Rocha-Gutiérrez, Samuel B. Pérez-Vega, Leslie R. Elías-Ogaz, Ivan Salmerón

**Affiliations:** 1 The Graduate School, Graduate Program in Food Technology, Autonomous University of Chihuahua, University Circuit 1, New University Campus, Chihuahua, Chihuahua C.P. 31125, Mexico; fzavala@uach.mx (F.J.Z.-D.d.l.S.); richy21021@gmail.com (R.C.-L.); pola_lerma@hotmail.com (L.P.L.-T.); brocha@uach.mx (B.A.R.-G.); sperez@uach.mx (S.B.P.-V.); lelias@uach.mx (L.R.E.-O.); 2 Departamento de Alimentos y Biotecnología, Facultad de Química, Universidad Nacional Autónoma de México, Circuito Exterior S/N, Coyoacán, Cd. Universitaria, Ciudad de México 04510, Mexico; panchote@unam.mx

**Keywords:** *Dasylirion* sp., sotol, spontaneous fermentation, microbial consortia, volatile metabolites, flavor attributes

## Abstract

In northern Mexico, the distilled spirit sotol with a denomination of origin is made from species of *Dasylirion*. The configuration of the volatile metabolites produced during the spontaneous fermentation of *Dasylirion* sp. must is insufficiently understood. In this study, the aim was to investigate the composition of the microbial consortia, describe the variation of volatile metabolites, and relate such profiles with their particular flavor attributes during the fermentation of sotol (*Dasylirion* sp.) must. Ascomycota was the phylum of most strains identified with 75% of total abundance. The genus of fermenting yeasts constituted of 101 Pichia strains and 13 Saccharomyces strains. A total of 57 volatile metabolites were identified and grouped into ten classes. The first stage of fermentation was composed of diesel, green, fruity, and cheesy attributes due to butyl 2-methylpropanoate, octan-1-ol, ethyl octanoate, and butanal, respectively, followed by a variation to pungent and sweet descriptors due to 3-methylbutan-1-ol and butyl 2-methylpropanoate. The final stage was described by floral, ethereal-winey, and vinegar attributes related to ethyl ethanimidate, 2-methylpropan-1-ol, and 2-hydroxyacetic acid. Our results improve the knowledge of the variations of volatile metabolites during the fermentation of sotol must and their contribution to its distinctive flavor.

## 1. Introduction

Mexico is a world-recognized country for the variety of its ancestral distilled beverages obtained from the fermentation of plants that belong to the family *Agavaceae,* such as *A*. *tequilina webber* for tequila, *A*. *angustifolia* and *A*. *potattorum* for mezcal, and *Dasylirion* species of the *Nolinacea* family for sotol. Dasylirion species are indigenous to the northern Mexican state of Chihuahua, where they grow in its canyons and desert. *Dasylirion* species commonly known as sotol or sereque were initially used by the native tribes of Chihuahua, Coahuila, Arizona, Texas, and New Mexico as food, for medical purposes, and in religious ceremonies. During the Spanish conquest with the introduction of the still, the distillation of fermented sotol must was implemented, improving the production of the distilled spirit sotol [1,2].

Sotol production methods have experienced slight modifications over the past two centuries. Initially, the plant is harvested when it reaches around six years of age after growing in adverse climate conditions, promoting the concentration of sugars in the heart of the plant. The “jimador” is the person in charge of cutting the plant, who, provided with an axe, removes the leaves of the pineapple, leaving intact the root of the plant so a new heart can grow in a couple of months. The pineapples are transported to the sotol production site, where they are cooked in an underground stone oven for around 12 h. Afterwards, the pineapples are ground in a mill, and the sotol must is let stand for a couple of days in open vessels or fermentation tanks. Then, water is added, and the sugars of the sotol must are biosynthetically converted to ethanol during a spontaneous fermentation process of around five to seven days. The fermented sotol must is then double distilled, and, finally, the alcohol concentration is adjusted from 35–55% before being bottled [1,3].

In 2002, the Mexican Institute of Industrial Property (IMPI) declared that sotol had an appellation of origin to the geographical region located in the Mexican states of Chihuahua, Coahuila, and Durango. In August of 2004, the secretary of the economy released the Mexican Official Norm NOM-159-SCFI-2004 “Alcoholic beverages-Sotol-Specifications and test methods” [4]. These regulations have prompted sotol in national and international markets. Although sotol has an international presence, there is limited information regarding the transformation process of sotol must during fermentation, the microorganisms involved, and the profiles of the volatile metabolites that give origin to the unique flavor of sotol.

It has been reported that sotol (*Dasylirion* sp.) is composed of complex sugars such as fructooligosaccharides, fructosyl polymers, and smaller molecules such as glucose and fructose. The concentration of reducing sugars in the extracts of cooked sotol pineapples can be around 31.9%, depending on the relation of water to mass weight of pineaaple used. During fermentation, reducing sugars are adjusted to around 10.5%, giving a concentration of soluble sugars of 10 °Brix, decreasing to 5 °Brix at the end of the fermentation. It has been described that, during the study of microorganisms present in sotol plant tissue and samples during the start and the end of sotol must fermentation, throughout morphological and biochemical tests, species of filamentous fungi, bacteria, and yeasts were identified [3]. Additionally, during the analysis of six commercial brands of the distillate sotol spirit, where the volatile compounds were assed, 25 chemical compounds were detected belonging to the chemical family of alcohols, volatile acids, aldehydes, esters, and polyphenols [5].

Previous investigations have presented an initial characterization of the alcoholic drink sotol, and a higher number of studies exist for other Mexican distilled spirits such as tequila and mezcal. In this study, the diversity of the microbial population during the spontaneous fermentation of sotol must was investigated and compared with a culture-independent (pyrosequencing) method. By sampling the fermentation process at different times, we were able to describe the composition and the distribution of volatile metabolites associated with the organoleptic characteristics of the spontaneous sotol must fermentation. Our study describes, for the first time, the great diversity of filamentous fungi and yeasts embraced in the artisanal fermentation of sotol must and the characterization of the volatile metabolites profiles during the process. These communities of microorganisms have a significant impact on the volatile metabolites produced during the fermentation and may influence the chemical composition of the distillate sotol spirit. With the information generated in this study, we can aid the understanding of the chemical transformation that occurs during sotol production.

## 2. Materials and Methods

### 2.1. Fermented Sotol Must Samples

Samples of the spontaneous fermentation of sotol must were collected daily, for six days, from an artisanal sotol production site located in the region of Aldama Chihuahua. The fermentation of sotol consisted of the following steps: pineapples of *Dasylirion* sp. collected in the southeast desert of Chihuahua were cooked in an underground stone oven for 12 h. Afterwards, the pineapples were milled, and then sotol grinded fractions were placed in open vessels that were let stand for two days. Subsequently, water was added (we defined this as day zero), and fermentation was ended at day five. The artisanal fermentation of the sotol must was performed with no agitation or temperature control, and the fermentation tanks were exposed to ambient temperatures (7–27 °C). The fermentation was finalized according to the sotol master, who decided this based on the minor production of bubbles in the fermentation tanks and the sour with mild acidic taste of the sotol must. Samples were withdrawn from the same fermentation tank during the process; these were placed in 500 mL sterile glass bottles and maintained at around 7 °C until reaching the laboratory, where samples were aseptically transferred to 50 mL falcon tubes and stored at −20 °C until analysis.

### 2.2. Enumeration of Microorganisms

The enumeration of dominant and culturable yeasts, filamentous fungi, and lactic acid bacteria (LAB) was performed based on colony-forming units (CFU), using decimal serial dilutions in saline solutions (0.9%) and plating onto culture media. Yeast and filamentous fungi were enumerated by using DifcoTM Yeast Extract-Peptone-Dextrose agar (Becton Dickinson, Mexico City, Mexico) and DifcoTM Potato Dextrose agar (BD, Mexico City, Mexico), respectively, and incubated at 25 °C. DifcoTM Lactobacilli MRS (de Man Rogosa Sharp and Merck, BD, Mexico City, Mexico) agar was used for LAB counts, and plates were incubated at 37 °C.

### 2.3. Microbial Consortium Identification and Data Analysis

Samples of 5 mL of the liquid fraction of fermenting sotol must at 0, 1, 2, 3, 4, 5, and 6 days were used for total DNA extraction by means of the method of phenol-chloroform according to Hoffman and Winston (1987) [6]. The quality and the quantity were checked by means of electrophoretic running from 25 nanograms of DNA. The ITS fragment was amplified with the markers 9 MID (TCTCTATGCG) and 11 MID (CATAGTAGTG) attached to the itsF oligo (3’-CTTGGTCATTTAGAGGAAGTAA-5′) and using the reverse ITS 4R (5’-TCCTCCGCTTATTGATATGC-3′) [7]. Equal concentrations of purified amplicons from the library were sent to Macrogen, Inc. (DNA Sequencing Service, Seoul, Korea) for pyrosequencing. The pyrosequencing was done with a Roche GS–FLX Titanium 454 pyrosequer (Roche, Mannheim, Germany). The resulting sequences were analyzed following the recommendations of the QIIME computer package in the tutorial “454 Overview Tutorial: de novo OTU picking and diversity analyses using 454 data”, making a mapping file and validating it with the program “validate_mapping_file.py” [8]. The resulting sequences were grouped together in operational taxonomic units (OTUs; defined as sequences clustered at a 97% probability level), and the chimeras were removed using the default indications of the Usearch and Uchime programs [9,10], and the nucleotide BLAST [11] was used to obtain the most similar sequences of GenBank.

### 2.4. Total Reducing Sugars (TRS) Determination

To measure total reducing sugars (TRS), the dinitrosalicylic acid method (DNS) by Bernfeld was used [12]. The absorbance of the samples was measured at 540 nm using a 96-well microplate reader model ELx808 (BioTek Instruments, Inc., Winooski, VT, USA). Sugar concentration was calculated by comparison with an external glucose calibration curve.

### 2.5. Volatile Metabolites Analysis by Headspace Solid-Phase Microextraction (HS-SPME)

Automated headspace solid-phase microextraction (HS-SPME) was performed using an auto-sampler CTC PAL (CTC Analytics, Zwingen, Switzerland). This was equipped with an SPME fibber/syringe holder, a temperature-controlled six-vial agitator tray, and a temperature-controlled chamber for the conditioning of the fibber. The SPME fibber material was polydimethylsiloxane/divinylbenzene (PDMS/DVB) (Supelco Inc., Bellefonte, PA, USA). Before the extraction of volatile metabolites, the fibber was conditioned according to manufacturer instructions. For each extraction, a working volume of 7 mL of fermented sotol must was placed into a 20 mL clear glass vial fitted with polypropylene hole screw caps and PTFE/silicone septum. The samples were equilibrated for 30 min at 50 °C in the temperature-controlled agitator tray. Afterwards, the SPME device was automatically inserted in the sealed vial through the septum, and the fibber was exposed to the volatiles of the headspace in the vial. Following this, the SPME fibber was immediately inserted into the gas chromatography (GC) injector, and the fibber was thermally desorbed for 20 min at 220 °C. The volatiles of fermented sotol must were identified using an Agilent 7890B gas chromatograph coupled to an Agilent 5975C mass selective detector (Palo Alto, CA, USA). Volatile metabolites were separated on a HP-INNOWAX MS capillary column (30 m × 0.25 mm ID; 1.0 µm film thickness, Agilent Inc., Santa Clara, CA, USA). The oven temperature program began with 3 min at 40 °C, followed by a 3 °C/min-rise to 120 °C, then a temperature ramp at 6 °C/min-rise to 200 °C and held at 200 °C for 20 min. Helium was used as the carrier gas at a flow rate of 1.2 mL/min. The quadrupole mass filter for mass spectrometric detection was operated at 150 °C in the electron impact mode (EI 70 eV). The ion source temperature was set at 220 °C, and the transfer line was set at 210 °C. The mass acquisition range was 40–400 *m*/*z*. The peaks were identified based on their fragmentation patterns using the NIST Mass Spectral Search Program version 2.0 (NIST, Washington, DC, USA). To perform the quantitative determination of volatile metabolites in fermented sotol at different days, samples were run in triplicate, and the integrated areas based on total ion chromatograms were normalized to areas of the internal standard and averaged. For this, 100 µL of 1-butanol (500 mg/L) was used as an internal standard.

### 2.6. Alcoholic Fermentation Efficiency

**Figure 1:**
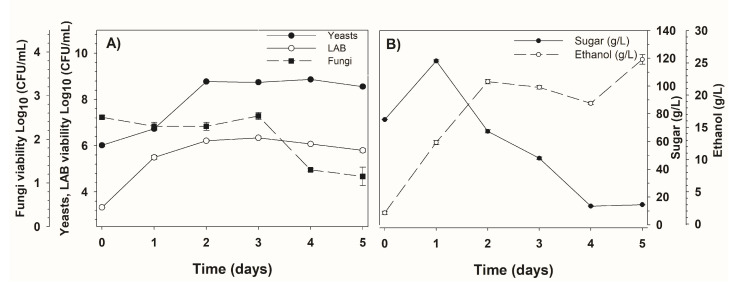
The evolution of principal microorganisms, sugar, and ethanol concentration during the fermentation of sotol (*Dasylirion* sp.) must. (**A**) Viability of filamentous fungi (■), total yeasts (●), and lactic acid bacteria (LAB) (○). (**B**) Profiles of sugar (●) and ethanol production (○).

The fermentation efficiency (FE) was estimated considering the ethanol present in the aqueous phase following the fermentation. Equation (1) defines FE, where ETOH is ethanol final concentration (g/L), TRS (g/L) is the sugar concentration at its highest level (as observed in Figure 1) that is due to the transfer of reducing sugars from the solid sotol particles to the aqueous phase, and 0.511 is the stoichiometric ethanol yield factor.

(1) $$\frac{ETOH}{0.511\cdot TRS}\cdot 100$$

### 2.7. Statistical Analysis

Data were analyzed statistically using a one-way analysis of variance (ANOVA) to determine whether significant differences (*p* < 0.05) existed. All data presented were mean values of replicates obtained from three independent samples of each fermentation time. A multivariate analysis of the volatile compounds at different times was conducted using a principal component analysis (PCA), performed with Minitab15 Statistical Software (Minitab Inc., State College, PA, USA). Factor loadings of the volatile compounds were combined into one bi-plot of PCA 1 and 2.

## 3. Results and Discussion

### 3.1. Fermentation Kinetics

The particular anatomy of sotol (*Dasylirion* sp.) delivers a rich growth medium that promotes the viability of yeasts, bacteria, and filamentous fungi, which complete the spontaneous fermentation of the sotol must [3]. Therefore, to better understand this interaction, the growth of the microbial population was analyzed during the spontaneous fermentation of sotol must, as depicted in Figure 1. A figure that illustrates the relative abundance of fungi and yeasts cell counts during fermentation can be found in the Supplementary Material (Figure S1). It was observed that yeasts were the most abundant microorganisms, followed by lactic acid bacteria (LAB) and then filamentous fungi. The growth trends for yeasts and LAB were similar throughout the fermentation. Yeast counts reached a maximum growth (8.77 log10 CFU/mL) after day two of fermentation. Similarly, for LAB, maximum growth (6.12 log10 CFU/mL) was achieved after day two. After that, the cell growth of yeasts was steady until the end of the fermentation on day five, where their growth began to decline, whereas the viable cells of LAB began to decline after day three. The numbers of yeasts and LAB reached levels of 8.55 and 5.79 log10 CFU/mL, respectively, at the end of the fermentation.

Our results showed that a symbiosis can exist among the microorganisms present in the spontaneous fermentation of sotol, which is in agreement with the observation presented in other studies of indigenous fermented beverages produced from *Agave* spp., such as tequila, bacanora [13,14], and mezcal [15]. In this context, spontaneous fermentation is a process originated and fulfilled by the combined action and/or succession of different species of yeasts, *Lactobacillales* and fungi, that can thrive together in a complex microbial consortium [16,17]. Yeasts start by acting upon sugars and consuming available oxygen and micronutrients, leading to an anoxic microenvironment, which promotes the production of ethanol. The anoxic conditions support the growth of LAB that produce lactic acid, which, in combination with the available ethanol, is metabolized by acetic acid bacteria (ABA) to produce volatile acetic acid. These metabolites and further biochemical changes give rise to significant flavor precursors [17,18]. The acidification of sotol must by LAB can also play a significant role in the process, as acidification can improve sugar extraction, fermentability, and nitrogen yield, improving color and flavor such as in acidic ales obtained by spontaneous fermentation [19].

The filamentous fungi presented a steady growth since day zero, suggesting that, when water was added to the sotol must (identified as day zero) after left to stand for two days, fungal growth was in an advanced stage. The maximum level of the filamentous fungi observed was 2.50 log10 CFU/mL, and after day three, the cell growth dropped, reaching a minimum level (1.15 log10 CFU/mL) at the end. Sotol filamentous fungi can play an important role in the viability of yeasts and LAB, as they are a source of fructotransferases [20] that act upon fructans, the natural occurring polysaccharides in sotol (*Dasylirion* spp.) [21]. It is then likely that fungi throughout the production of fructotransferases participate in the initial phase of sotol must fermentation, hydrolyzing fructans found in the cellular structure and liberating fructose along with glucose, which aid the initial growth of yeasts and LAB. Additionally, fungi belonging to the genera *Aspergillus* and *Penicillium* have been reported to produce a combination of endo- and exo-inulinases, that release oligosaccharides along with fructose from inulin-rich plant extracts [22]. It is then important to perform further studies in order to evaluate the hydrolytic activities of fungal strains isolated from sotol plant.

Figure 1 shows that the initial concentration of sugars was around 75.84 g/L; this increased to 118.1 g/L after 24 h due to the transfer of these from the sotol fibbers to the liquid medium. Fermentable sugars decreased as a consequence of the LAB and the yeasts biosynthetic demands during their growth. Sugar consumption was not completed, leaving around 14.45 g/L of residual sugars in the culture medium at day five of the fermentation. The value of ethanol at the end of the fermentation was 25.53 g/L, and based on sugar consumption, the fermentation efficiency was 42.3%. Previous studies of artisanal fermentation of *Agave* spp. for mezcal and tequila report alcoholic fermentation efficiency values of 34.9–39% in mezcal [23] and 33–34% in tequila [24]. Similarly, in these works, there was an activity of mixed strains of *Saccharomyces* and non-*Saccharomyces* strains, and the consumption of sugars was not complete, leaving sugars in the fermenting media.

Yeast are capable of producing ethanol with high yields when optimum parameters are established; unfortunately, aerial or external contamination is a factor that disturbs the fermentation system [17]. This factor makes spontaneous fermentations performed in open tanks susceptible to microbial contamination, such as in Lambic breweries where the presence of enterobacteria in the initial stage has been attributed to contamination from the air [19]. LAB contamination can raise organic acid values, altering ethanol production, and a concentration of lactic acid around 0.9% (*w*/*v*) prior to fermentation can decrease yeast growth and affect ethanol production [25]. Thus, this is an issue that should be further studied, as we detected a significant growth of LAB by microbial culture. Thus, bacteria strains can be identified by non-culture-based techniques in subsequent works. Moreover, other reasons that can induce a low fermentation yield in beverages produced with agave juice are nitrogen, vitamins, or oxygen deficiencies, as the thermal processing of agave reduces the assimilable nitrogen content, affects vitamin values, and can render insignificant levels of oxygen [26]. Nitrogen levels in the sotol medium should be further studied, as the presence of amino acids in the medium increases the ability of rapid synthesis of degraded proteins as glucose transporters, which allow the yeasts to achieve complete fermentation in the particular case of non-*Saccharomyces* strains [27]. However, *Saccharomyces cerevisiae,* which is considered as glucosophilic due to its sugar transport mechanisms, exhibits greater fermentation capabilities attributed to its ability to metabolize both inorganic and organic nitrogen sources from the fermenting media [28].

### 3.2. Microbial Identification

**Figure 2:**
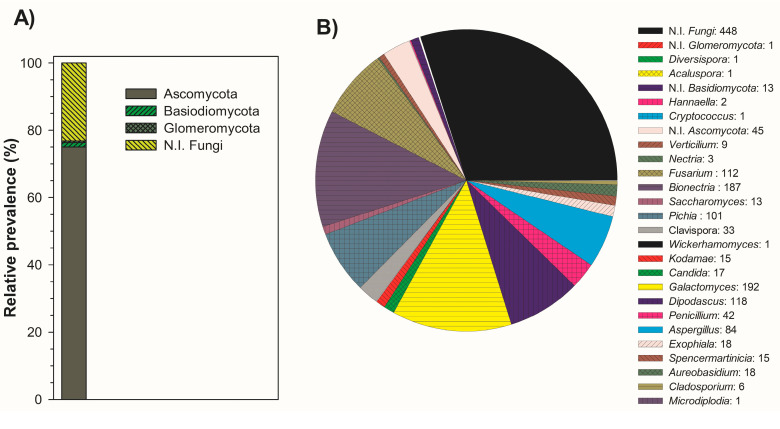
Relative abundance at the phylum level and counts of filamentous fungi and yeasts in fermented sotol (*Dasylirion* sp.) must. (**A**) Microbial groups with relative prevalence. (**B**) Filamentous fungi and yeast diversity: number of strains.

Fermentation is an old technique used by different civilizations for the elaboration and the preservation of foods. Records of different fermentation methods applied to a variety of raw materials demonstrate that this practice has been applied since 6000 BC in the Middle East region [29]. This metabolic process is critical in food elaboration, affecting the organoleptic quality of the raw material [30]. During the production of many autochthonous alcoholic beverages, the participation of microbial consortiums converts the fermentable sugars present in the media into ethanol and secondary metabolites of important flavor attributes [31]. Thus, different species of filamentous fungi and yeasts can participate in spontaneous fermentations. In Figure 2, we present the fungal diversity and the genus of microorganisms identified in the samples of fermented sotol must, and data showing the application of some of the species recognized in this study can be found in the Supplementary Materials (Table S1). In Figure S2 of this section, we present photos of DNA extraction from samples of spontaneous fermented sotol must, and in Figure S3 we present the amplification of DNA by PCR, from sotol samples. It was observed that Ascomycota is the phylum of most microorganisms identified with 1449 strains, followed by Basidiomycota with 26 strains, Glomeromycota with nine strains, and 448 strains of not identified fungi (Figure 2).

The genus of the identified strains was conformed mostly by *Galactomyces* (192), *Bionectria* (187), *Dipodascus* (118), *Fusarium* (112), *Pichia* (101), and *Aspergillus* (84), and of *Saccharomyces,* only 13 strains were recognized (Figure 2).

In previous works, species of *Galactomyces* were identified as the dominant yeasts [32], and particularly the species *Galactomyces geotrichum*, identified in fermented sotol must (see Table S1), has been attributed to possess the metabolic pathways to produce significant aromatic metabolites [33]. *Dipodascus,* a group of yeasts described as extremophiles, were reported in the fermentation of coffee beans performed in open tanks and are vulnerable to contamination from air, human contact, and insects [34]. Additionally, species of *Dipodascus* have been identified in the tissue of cactus indigenous to the Americas, specifically in Southern Arizona [35], a region that shares geographic and environmental conditions with the Chihuahua desert, which is the native habitat of sotol (*Dasylirion* spp).

Another group of yeasts identified in fermented sotol must was *Pichia* sp., which has the metabolic ability to consume sotol must sugars and is capable of adapting to the stress factors of sotol processing. In a previous work about the production of potential volatile flavor metabolites isolated from the spontaneous fermentation of coffee beans pulp, strains belonging to the species *Pichia kudriavzevii* were the most aroma-producing yeasts [36]. This Pichia species was identified during sotol must fermentation (see Table S1). Furthermore, these yeast species have been reported in several spontaneous fermentation processes, such as in Ghanaian and Ivorian cocoa beans fermentation, where the dominance of *Pichia kudriavzevii* strains is attributed to their ability of adaptation to stressful environmental conditions, such as elevated ethanol concentrations, high temperatures, and low acidity levels [36]. Moreover, these species can be found in foods containing high levels of sugars, such as maple syrup, fruit juices, dry fruit, jams, honey, and fermented honey [37]. Furthermore, *Pichia* species (main non-*Saccharomyces* yeasts) have been identified during the fermentation of alcoholic beverages [38,39] and in distilled spirits. They contribute to the aroma of these by the production of metabolites such as esters, higher alcohols, acids, terpenoids, and aldehydes [40]. Therefore, this yeast genus encompasses the biosynthetic ability to play a significant role during the spontaneous fermentation of sotol must.

In the process of alcoholic beverages, *Saccharomyces cerevisiae* is the yeast species of globally recognized application for the transformation of sugary substrates to beverages of unique flavor and aroma characteristics. In fermentations for the production of wine, beer, whiskey, tequila, mezcal, and other distilled spirits, *S*. *cerevisiae* converts the sugary substrates into ethanol, carbon dioxide, and various secondary metabolites, which convene to give a particular flavor in the final spirit. In our work, it was observed that the yeast species *S*. *cerevisiae* was present in low strain numbers in the fermented sotol must (Figure 2 and Table S1); however, its contribution to the sensory quality of sotol is of great importance. It is reported that, in spontaneous fermentations, *S. cerevisiae* is the dominant species as ethanol concentration increases. Nevertheless, this yeast is absent or present in low numbers at the start of the process, leading to the foundation that the fermentation production site is the primary source for this yeast [31]. Additionally, the presence of the dominant *S*. *cerevisiae* species is attributed to their adaptation capability to stressful environmental conditions, such as increasing ethanol concentrations and temperature changes during the fermentation [41].

Another group of microorganisms identified in the fermentation samples of sotol must was the filamentous fungi shown in Figure 2 and Table S1, of which the *Aspergillus* genus stands out due to its reported presence in artisanal fermented processes. The employment of *Aspergillus* species is common in the production of alcoholic beverages formulated with cellulose-containing substrates. These cultures hold cellulose-degrading enzymes that improve the breakdown of polysaccharides and increase the ethanol yield of *Saccharomyces cerevisiae* strains [42]. Moreover, fermentation with fungal strains (individually and consortium) of *Aspergillus* has been employed in the degradation of lignocellulose substrates such as raw sugarcane bagasse and wheat bran. These fermentations show the production of exoglucanase, B-xylosidase, endoglucanase, and manganese peroxidase [43]. The hydrolytic capacity of filamentous fungi enzymes to enhance sensory characteristics has been studied, showing significant levels of enzymes (xylanase, cellulose, amylase, and pectinase) produced by *Aspergillus* versicolor strains [44], whereas *Aspergillus flavus* strains are important inulinase production microorganisms [45]. Furthermore, these strains exhibit the ability to produce ascorbic acid, a metabolite that can improve the growth of other cultures during consortium fermentations [46].

The genus *Fusarium* is a fungi that has the biosynthetic pathways to contribute to the flavor attributes of fermented beverages, as this produces short-chain fatty acids of important flavor attributes such as 4-decanolide [47], which exhibits fruity and peach-like aroma attributes [48]. The fungal genus of *Bionectria* produces*,* rather than flavor characteristics, metabolites exhibiting a broad range of antibacterial, antifungal, and anti-dermatophytic activity [49]. For instance, the species *Bionectria ochroleuca* has been reported to produce bioactive polyketide glycosides that inhibit *Candidad albicans* biofilm formation [50]. Furthermore, this species reports showing fibrinolytic enzyme activity [51].

During the spontaneous fermentation of sotol must, a complex microbial consortium interacts to give the physicochemical and the sensory attributes that distinguish the alcoholic spirit sotol. As a result, filamentous fungi, yeasts, and bacteria play significant roles in producing significant flavor attributes. Changes in temperature, pH, and an increase of ethanol concentration affect the growth of certain microorganisms but also promote the viability of others, affecting the metabolites produced during the spontaneous fermentation process of sotol must.

### 3.3. Variation of Volatile Metabolites Profiles During Sotol Must Fermentation

**Figure 3:**
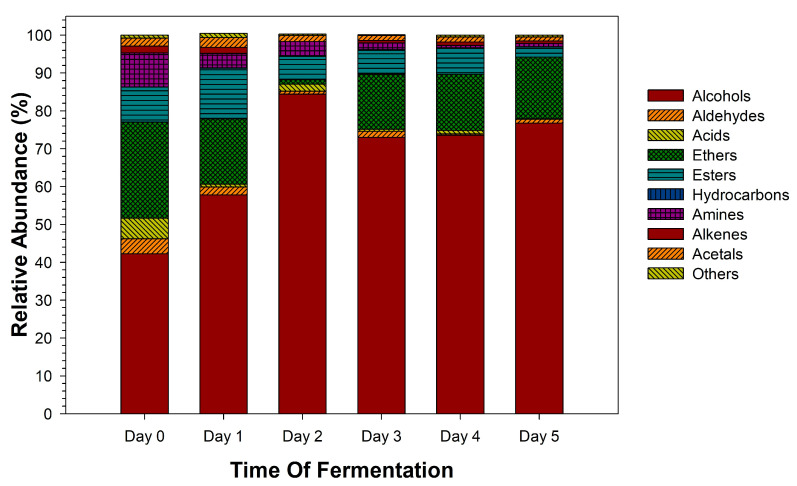
The relative abundance of volatile compounds among different chemical classes in fermented sotol (*Dasylirion* sp.) must at different days of the process.

The transformation of the volatile profiles during the fermentation of sotol must is an important feature that shapes the organoleptic attributes of the final distilled spirit. The chromatograms of the volatile metabolites detected in fermented sotol (*Dasylirion* sp.) must are shown in Figure S4. Their retention times and identification are exhibited in Table 1, and the classifications of the metabolites by chemical groups are shown in Figure 3. A total of 57 compounds were identified and grouped into ten classes based on the chemical properties as follows: alcohols (9), aldehydes (6), acids (7), ethers (3), esters (14), hydrocarbons (3), amines (6), alkenes (2), acetals (2), and others (5). Until today, there have been no reports about volatiles profiles in fermented sotol must. De la Garza et al. (2010) reports a significant effect on the organoleptic characteristics of the distillate sotol given by alcohols, volatile acids, aldehydes, esters, and polyphenols. Lachenmeier et al. (2006) performed a gas chromatography (GC) and ion chromatography (IC) analysis to study Mexican spirits such as sotol, where the identification of alcohols, aldehydes, and esters was reported, and the anionic profile of sotol exhibited a high content of nitrate.

Of the ten chemical groups used to classify the volatile metabolites produced during fermentation (Figure 3), alcohols was the most predominant category; secondly, the group of ethers, followed by esters, then amines, and minimum levels of acids and aldehydes were observed. These volatile metabolites derive from the alcoholic fermentation by yeasts and other microorganisms present in sotol must, which metabolize carbohydrates, fatty acids, amino acids, and other organic components. Thus, likewise, there is the primary production of ethanol by yeasts of other volatile metabolites, such as aldehydes, ketones, organic acids, and higher alcohols [52]. Yeasts can produce esters by the reaction of fatty acids when ethanol is produced in excess [53]. Additionally, by malolactic fermentation (MLF), LAB can affect the flavor properties due to compositional changes, including the metabolism of amino acids, polyols, hydrolysis of glycosides, and ester synthesis [54]. LAB also has an essential role in the metabolism of unsaturated fatty acids generating precursors of γ-lactones, which supply a sweet and fatty flavor to alcoholic liquors [55]. Finally, other compounds such as terpenoids might come from the plant, and many others are produced during the cooking process [53].

Higher alcohols play an important role in the flavor of fermented beverages; in our study, C5 and C8 alcohols were abundant in fermented sotol must. In particular, the identification of 3-methylbutan-1-ol (banana, pungent, and pear attributes) and octan-1-ol (fresh and green leaves attributes) was performed (Table 1). Other alcohols identified were 3,4-dimethylpentan-1-ol and 2-methylpropan-1-ol, which exhibit petrol and ethereal-winey aroma qualities, respectively. The occurrence of these metabolites was due to the activity of yeasts, amino acids in the substrate, and environmental conditions [62]. Yeasts produce fusel alcohols from α-keto acids through the metabolism of amino acids by the Ehrlich pathway, thus branched-chain alcohols such as 3-methylbutan-1-ol, detected in sotol must could be produced after the degradation of the branched-chain amino acid leucine [63]. Thus, it is important that, as with the *Saccharomyces* species and environmental conditions, the amounts and the types of amino acids present in the sotol must affect the production of higher alcohols that exhibit different aroma characteristics [63,64].

In particular, esters can be considered the most important class of flavor compounds detected during the fermentation of sotol must; namely, butyl isobutyrate was the most abundant ester at the beginning of the process (day zero) and decreased during fermentation until the process was stopped (day five). Bis(trimethylsilyl) oxalate, octyl ester, and butyl octanoate of green, ethereal, and herbal flavor properties were detected only at the start of the process. Meanwhile, ethyl acetate and ethyl octanoate of fruity and sweet flavor characteristics were identified in the middle and the final stages of the fermentation. Finally, ethyl ethanimidate of floral notes was detected only at the end of the sotol must fermentation. These metabolites with pleasant flavor properties are important in the perceived flavor of the final distilled spirit. The origin of these compounds can be attributed to the esterification reactions between organic acids and alcohol during the fermentation and aging process [65]. Among these metabolites, ethyl acetate is the main ester that occurs in fermented products and their distillates, and commonly commercial strains of *Saccharomyces cerevisiae* give rise to this compoun and other acetate esters; however, great interest has emerged in the use of non-*Saccharomyces* species due their greater potential for the production of novel aromas [66]. Therefore, due to the spontaneous fermentation of sotol must, it is clear that *S*. *cerevisiae* strains and non-*Saccharomyces* species contribute to the formation of ethyl ester and butyl isobutyrate, which exhibit important organoleptic properties such as a strong fruity odor and sweet pineapple taste [67].

The volatile acids presented a profile that decreased during the fermentation. As with butanoic acid, butyl ester (with cheese and rancid properties), and formic acid, ethenyl ester (with floral, honey properties) had its highest abundance at the start of the fermentation, and then its values dropped during the fermentation process. Hydroxyacetic acid that exhibits vinegar-like organoleptic properties was detected from the middle part of the fermentation to the end of the process. These acids are composed mainly of short-chain acids and medium-chain saturated acids, where the first ones are produced as metabolic by-products during the alcoholic fermentation, and the second ones are considered as intermediates of long-chain fatty acids biosynthesis [64]. Additionally, the presence of these compounds is attributed to the metabolism of non-*Saccharomyces* species, which tend to increase acid production under aerobic conditions due to their oxidative metabolism affecting the formation of ethanol, glycerol, and organic acids. The activity of aerobic acetic acid bacteria and some non-*Saccharomyces* species considered as spoilage yeasts can metabolize ethanol and produce acetic at concentrations that can affect the flavor attributes in fermented beverages [63]. Additionally, the composition of the fermenting medium can modulate the formation of these compounds of vinegar and sour attributes [68].

### 3.4. PCA Analysis of Aroma Compounds Variation During Sotol Must Fermentation

**Figure 4:**
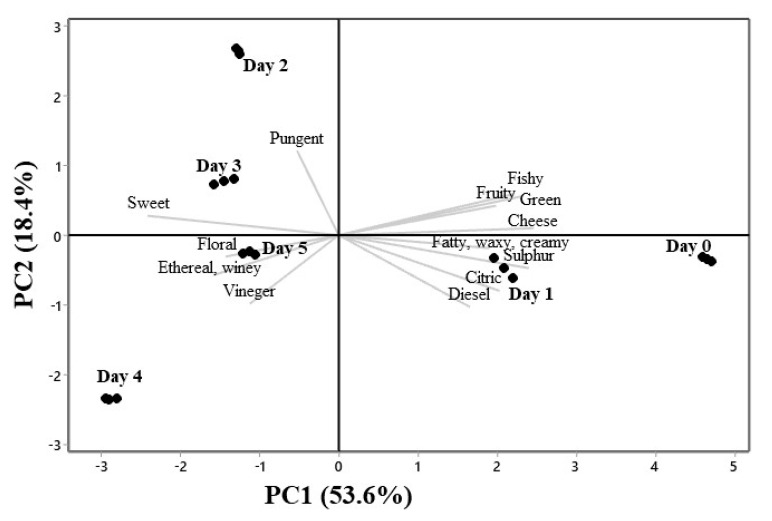
Principal component analysis bi-plot of the composition of flavor attributes of the volatile compounds detected, as correlation loadings in fermented sotol (*Dasylirion* sp.) must at different days (●) of the process as scores.

PCA analysis was performed based on the aroma properties of volatile metabolites reported in Table 1 to investigate the effects of fermentation time on the production of aroma compounds. As exhibited in Figure 4, the first two principal components explained 72% of the total variance (PC1 53.6%, PC2 18.4%, and PC3 16.1% result not shown). There was a clear separation of the aroma compounds of day zero and day one from the rest of the fermentation days, indicating significant changes in the aroma compounds during the fermentation process. In particular, day one was characterized by the presence of metabolites with attributes such as diesel, citric, sulphur, fatty, waxy, and creamy. Day two and day three were described by the attributes pungent and sweet. The final part of the fermentation, represented by day five, was characterized by the attributes floral, vinegar, ethereal, and winey. It was evident that the flavor attributes of the middle part of the fermentation represented by day two and day three and the aroma attributes of the final part of the fermentation described in day four and day five were separated based on PC2. PCA results suggest that the analysis method and data processing are reliable for separating the aroma attributes of the sotol must fermentation, being an accurate approach to discriminate the changes of the volatile metabolites due to the biosynthetic activities of the fermenting microbial consortia.

## 4. Conclusions

The current study investigated the microorganisms present in the spontaneous fermentation of sotol must and assessed the variation of volatile metabolites occurring. Results obtained by non-culture dependent methods exhibited the presence of a complex microbial consortium of filamentous fungi and yeasts, which interact in a symbiosis, liberating a wide range of volatile metabolites that vary according to changes of the fermentation environment. Through the application of the PCA analysis, it was possible to identify those alcohols, acids, and aromatic metabolites of particular flavor attributes, which could be further used to depict the fermentation stage of sotol must. Finally, the results observed in this study contribute to the understanding of the microbial consortia present in the fermentation process of sotol must and improve the knowledge of the variation of volatile metabolites occurring.

## Figures and Tables

**Table 1:** The concentration of volatiles in fermented sotol (*Dasylirion* sp) must (mg/L). Mean ± STD (n = 3).

Compound	RT (Min)	Flavor Descriptor ^a^	Days of Fermentation					
			0	1	2	3	4	5
Alcohols								
2-methylpropan-1-ol	6.86	Ethereal, winey	-	28.11 ± 2.38	-	-	33.45 ± 2.41	-
2-ethylcyclobutan-1-ol	9.64		-	-	-	18.82 ± 0.06 ^a^	18.43 ± 0.35 ^a^	-
3-methylbutan-1-ol	10.81	Fruity, pungent	-	39.62 ± 5.14 ^b^	61.31 ± 3.18 ^a^	57.63 ± 1.22 ^a^	-	-
3-bromopentan-2-ol	16.38		-	-	-	9.48 ± 0.18	-	-
6-amino-2-methylheptan-2-ol	20.91		-	8.81 ± 0.23	-	-	-	-
octan-1-ol	22.33	Green leaves	35.28 ± 0.53 ^b^	44.76 ± 1.53 ^a^	38.60 ± 3.73 ^b^	36.80 ± 1.26 ^b^	9.56 ± 0.18 ^d^	29.59 ± 0.26 ^c^
								
(2R)-6-methyl-2-[(1R)-4-methylcyclohex-3-en-1-yl]hept-5-en-2-ol	23.62	Diesel	-	10.46 ± 0.10 ^c^	12.94 ± 1.58 ^b^	18.18 ± 0.76 ^a^	18.06 ± 0.57 ^a^	-
(+)-Alpha-bisabolol	41.02		-	-	9.35 ± 0.24	-	-	-
(2R,3R,4R,5S)-hexane-1,2,3,4,5,6-hexol	45.85	Sweet	-	-	10.75 ± 0.38	-	-	-
Aldehydes								
butanal	2.39	Cheese, fruity	66.92 ± 7.37 ^a^	61.78 ± 12.21 ^a^	29.23 ± 1.44 ^b^	18.51 ± 2.21^b^	-	31.69 ± 1.20 ^b^
2-ethyl hexanal	4.82		24.35 ± 1.53	-	-	-	-	-
3-hexylimino-2-nitropropanal	10.12		-	-	-	-	-	10.39 ± 0.20
1,6,6-trimethylbicyclo [2.1.1]hexane-5-carbaldehyde	26.82			10.88 ± 0.57 ^a^	-	-	-	10.47 ± 1.11 ^a^
3-[(*E*)-hydroxyiminomethyl]phenol	40.82		-	-	-	36.67 ± 2.78 ^a^	13.99 ± 0.89 ^b^	11.03 ± 0.63 ^b^
3,6-dimethyl-3,3*a*,4,5-tetrahydro-2*H*-indene-1-carbaldehyde	45.86		-	-	-	10.51 ± 0.41	-	-
Acids								
2-(carbamoylamino)-2-oxoacetic acid	4.30		11.71 ± 0.20	-	-	-	-	-
acetohydrazide	4.33		-	-	-	-	8.71 ± 0.16	-
2-hydroxyacetic acid	5.12	Vinegar	-	-	10.89 ± 0.34 ^a^	10.33 ± 0.39 ^a^	10.51 ± 0.13 ^a^	-
(2*R*,3*R*)-2-amino-3-hydroxybutanoic acid	8.02		-	-	-	10.68 ± 0.31 ^a^	10.74 ± 0.44 ^a^	-
1,3,4-trihydroxy-5-oxocyclohexane-1-carboxylic acid	21.17		-	-	-	16.06 ± 12.01	-	-
2-(propan-2-ylcarbamoylamino)acetic acid	27.19		-	11.06 ± 0.55	-	-	-	-
2-(carbamoylamino)-2-oxoacetic acid	40.81		-	-	-	-	-	13.75 ± 0.12
Ethers								
1-butoxybutane	3.43	Diesel	473.13 ± 29.80 ^a^	432.21 ± 17.16 ^a,b^	24.96 ± 1.58 ^d^	386.95 ± 26.06 ^b,c^	334.51 ± 19.23 ^c^	476.67 ± 22.96 ^a^
1-[(*E*)-but-1-enoxy]butane	4.66	Sulphur	41.20 ± 1.18 ^b^	49.94 ± 3.94 ^a^	12.05 ± 1.25 ^d^	20.79 ± 1.90 ^c^	19.17 ± 3.25 ^c^	17.52 ± 0.99 ^c,d^
5-ethyl-2,4-dipropyl-1,3-dioxane	17.83	-	15.28 ± 1.04 ^ab^	17.64 ± 1.11 ^a^	10.34 ± 0.52 ^c^	14.51 ± 1.54 ^b^	-	12.73 ± 1.07 ^bc^
Esters								
ethenyl formate	1.52	Floral	-	156.64 ± 27.28 ^a^	101.65 ± 12.71 ^b^	91.84 ± 7.31 ^b^	120.89 ± 6.83 ^a^	79.94 ± 13.68 ^b^
ethyl acetate	2.48	Fruity, pineapple	-	-	19.86 ± 1.38 ^b^	17.92 ± 0.20 ^c^	16.48 ± 0.19 ^d^	25.63 ± 0.13 ^a^
ethyl ethanimidate	5.09	Floral	-	-	-	-	-	20.97 ± 0.35
butan-2-yl nitrite	7.45		-	-	-	10.46 ± 0.24 ^b^	10.02 ± 0.34 ^b^	11.27 ± 0.33 ^a^
butyl 2-methylpropanoate	8.18	Fruity	94.99 ± 2.58 ^a^	63.36 ± 5.21 ^b^	17.75 ± 0.57 ^d^	59.30 ± 1.22 ^b,c^	50.85 ± 7.07 ^c^	50.51 ± 4.64 ^c^
pentyl prop-2-enoate	10.79		22.13 ± 1.74	-	-	-	-	-
butyl butanoate	10.96	Cheese	107.56 ± 5.61 ^b^	142.67 ± 6.72 ^a^	40.13 ± 3.15 ^c^	109.97 ± 12.82 ^b^	-	106.3 ± 4.60 ^b^
ethyl 2-hydroxypropanoate	16.36		-	-	-	-	12.64 ± 0.24 ^b^	17.14 ± 1.30 ^a^
bis(trimethylsilyl) oxalate	18.37	Green	17.99 ± 0.58	-	-	-	-	-
ethyl octanoate	19.88	Fruity, winey	-	24.20 ± 2.56 ^a^	14.49 ± 1.07 ^b^	-	-	17.55 ± 0.39 ^c^
octyl 2-methylpropanoate	23.39	Vinegar	-	14.65 ± 0.62	-	-	-	-
octyl ester	23.41	Waxy	14.73 ± 1.41	-	-	-	-	-
thanedioic acid, bis(trimethylsilyl) ester	25.45		75.12 ± 12.50	-	-	-	-	-
butyl octanoate	26.99	Creamy	-	13.35 ± 1.21	-	-	-	-
Hydrocarbons								
4-methyl-2,3,4,5,6,7-hexahydro-1*H*-indene	22.91	Fruity	-	10.49 ± 0.42 ^a^	10.53 ± 0.56 ^a^	10.19 ± 0.26 ^a^	-	10.05 ± 0.35 ^a^
3,5-dimethyl-1,2,4-trioxolane	26.40		-	-	-	-	-	9.83 ± 0.15
4,4-dimethyl-8-methylidene-1-oxaspiro[2.5]octane	26.82		-	-	-	10.65 ± 0.38	-	-
Amines								
2-(aziridin-1-yl)ethanamine	1.26	Fishy	126.19 ± 26.0 ^a^	94.99 ± 3.62 ^a^	102.99 ± 10.89 ^a^	50.21 ± 2.05 ^b^	-	-
octan-2-amine	1.53		65.53 ± 10.29	-	-	-	-	-
dimethyl(trimethylsilyloxy)silicon	2.22		-	-	12.44 ± 0.55 ^b^	19.95 ± 0.42 ^a^	19.54 ± 0.50 ^a^	19.75 ± 0.99 ^a^
*N*-methyl-*N*-(2-methylpropyl)nitrous amide	5.94		-	-	-	-	-	22.01 ± 2.0
(2*R*,3*R*)-2-amino-3-hydroxybutanoic acid	7.99		-	18.84 ± 1.17	-	-	-	-
3,4-diamino-4-oxobutanoic acid	19.90		11.32 ± 0.46 ^b^	-	-	10.45 ± 0.18 ^c^	16.17 ± 0.74 ^a^	-
Alkenes								
1-[(*E*)-but-2-enoxy]butane	5.84	Diesel	43.84 ± 1.72 ^b^	52.40 ± 3.90 ^a^	-	23.17 ± 1.68 ^c^	25.95 ± 1.11 ^c^	27.41 ± 0.54 ^c^
1,4-dimethoxybenzene	39.20	Sweet, green	-	-	-	9.82 ± 0.21 ^b^	9.56 ± 0.35 ^b^	10.78 ± 0.25 ^a^
Acetals								
4-methyl-2-pentyl-1,3-dioxolane	7.23	Fruity, sweet	41.18 ± 3.51 ^a^	40.76 ± 4.18 ^a^	38.17 ± 4.74 ^a^	35.66 ± 2.94 ^a^	33.45 ± 2.41 ^a^	33.32 ± 1.79 ^a^
2-butyl-4-methyl-1,3-dioxolane	7.89	Fatty	15.98 ± 1.02 ^b,c^	46.98 ± 1.55 ^a^	17.09 ± 1.67 ^b^	15.26 ± 0.47 ^bc^	13.45 ± 1.27 ^c^	14.40 ± 0.63 ^bc^
Other compounds								
3,3-dimethyldiaziridine	4.82		-	27.57 ± 1.93 ^a^	-	11.31 ± 0.85 ^b^	12.59 ± 1.32 ^b^	11.28 ± 0.50 ^b^
1-methyl-4-prop-1-en-2-ylcyclohexene	9.66	Citric, lemon	23.58 ± 1.67 ^a^	17.20 ± 1.14 ^c^	-	-	14.61 ± 0.71 ^c^	20.39 ± 0.85 ^b^
2-hydroxypropanamide	13.84		-	-	10.32 ± 0.71	-	-	-
prop-2-enylurea	19.44		-	-	9.75 ± 0.09	-	-	-
1-methylpyrazole-4-carbaldehyde	25.58		-	-	11.09 ± 0.42	-	-	-

^a^ Reference: [56,57,58,59,60,61]. Data show means compared by one-way analysis of variance (ANOVA), where significant differences of *p* < 0.05 were compared by Tukey’s multiple comparison test, with significant differences within a row shown by different letters ^a b c d^.

## Supplementary Materials

The following are available online at https://www.mdpi.com/2218-273X/10/7/1063/s1, Figure S1. Relative abundance of yeasts and fungi counts in fermented sotol (*Dasylirion* sp.) must at different days of the process, Figure S2. DNA Extraction from samples of spontaneous fermented sotol must: Agarosa gel 1%, dyed with ethide bromide, MWM, molecular weight marker ladder 1Kb Invitrogen, Figure S3. Amplification of DNA by PCR from, sotol samples, Figure S4. Chromatograms of volatiles from fermented sotol (*Dasylirion* sp.) must at different days of the process, Table S1. Identified species in fermented sotol (*Dasylirion* sp) must of significant fermentation applications.
