# Supplementary material for: Understanding the Biosynthetic Changes that Give Origin to the Distinctive Flavor of Sotol: Microbial Identification and Analysis of the Volatile Metabolites Profiles During Sotol (Dasylirion sp.) Must Fermentation
Source: Biomolecules. 2020 Jul 16;10(7):1063. doi: 10.3390/biom10071063 (PMC7408159; doi:10.3390/biom10071063)
Supplement: Supplementary file 1 [file biomolecules-10-01063-s001.pdf]

biomolecules

Supplementary Materials

Article

Understanding the biosynthetic changes that give origin to the distinctive flavour of Sotol: Microbial identification and analysis of the volatile metabolites profiles during sotol (*Dasylirion* sp.) must fermentation

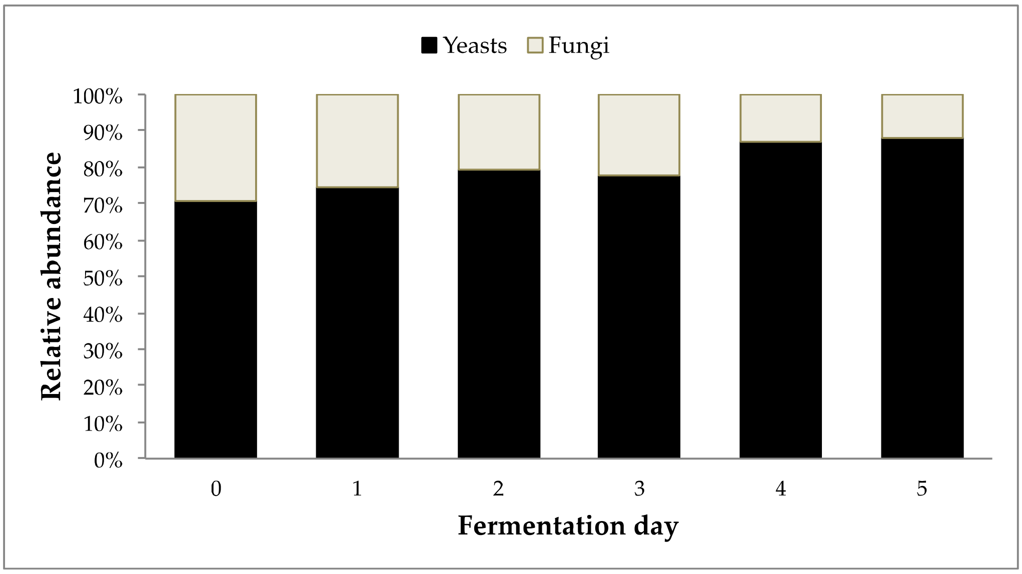

Figure S1. Relative abundance of yeasts and fungi counts in fermented sotol (*Dasylirion sp.)* must at different days of the process.

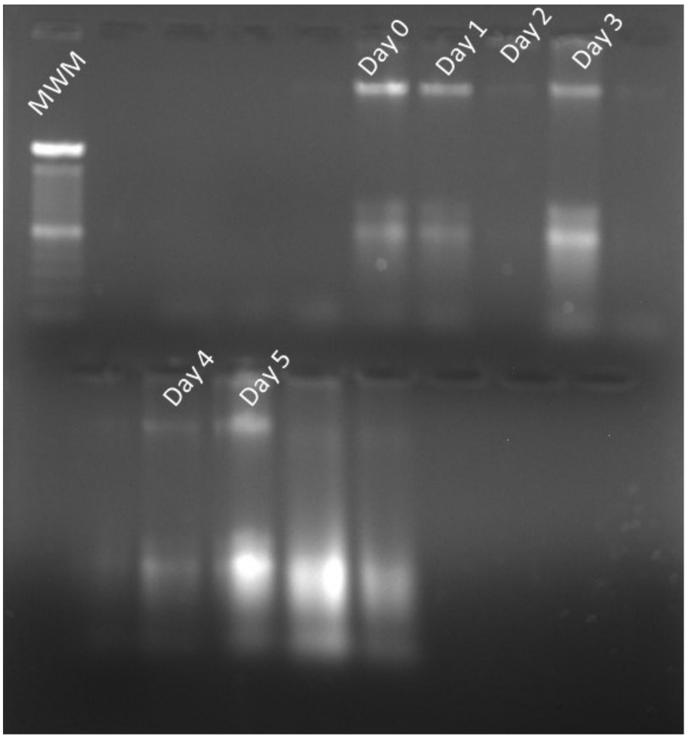

Figure S2. DNA Extraction from samples of spontaneous fermented sotol must. Agarosa gel 1%, dyed with ethide bromide. MWM, molecular weight marker ladder 1Kb Invitrogen.

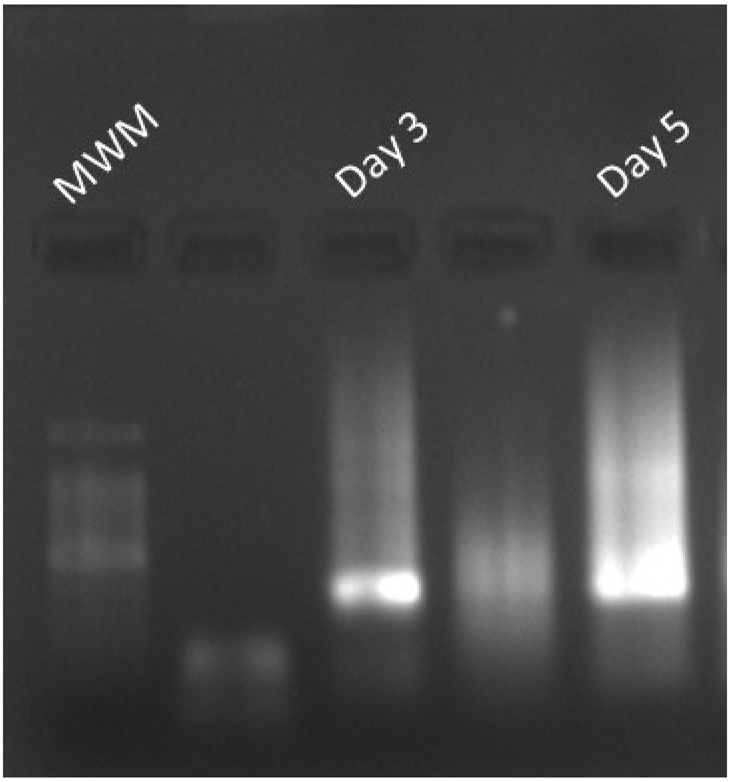

Figure S3. Amplification of DNA by PCR, from sotol samples. Agarosa gel 1%, dyed with ethide bromide. MWM, molecular weight marker ladder 100 bp Invitrogen.

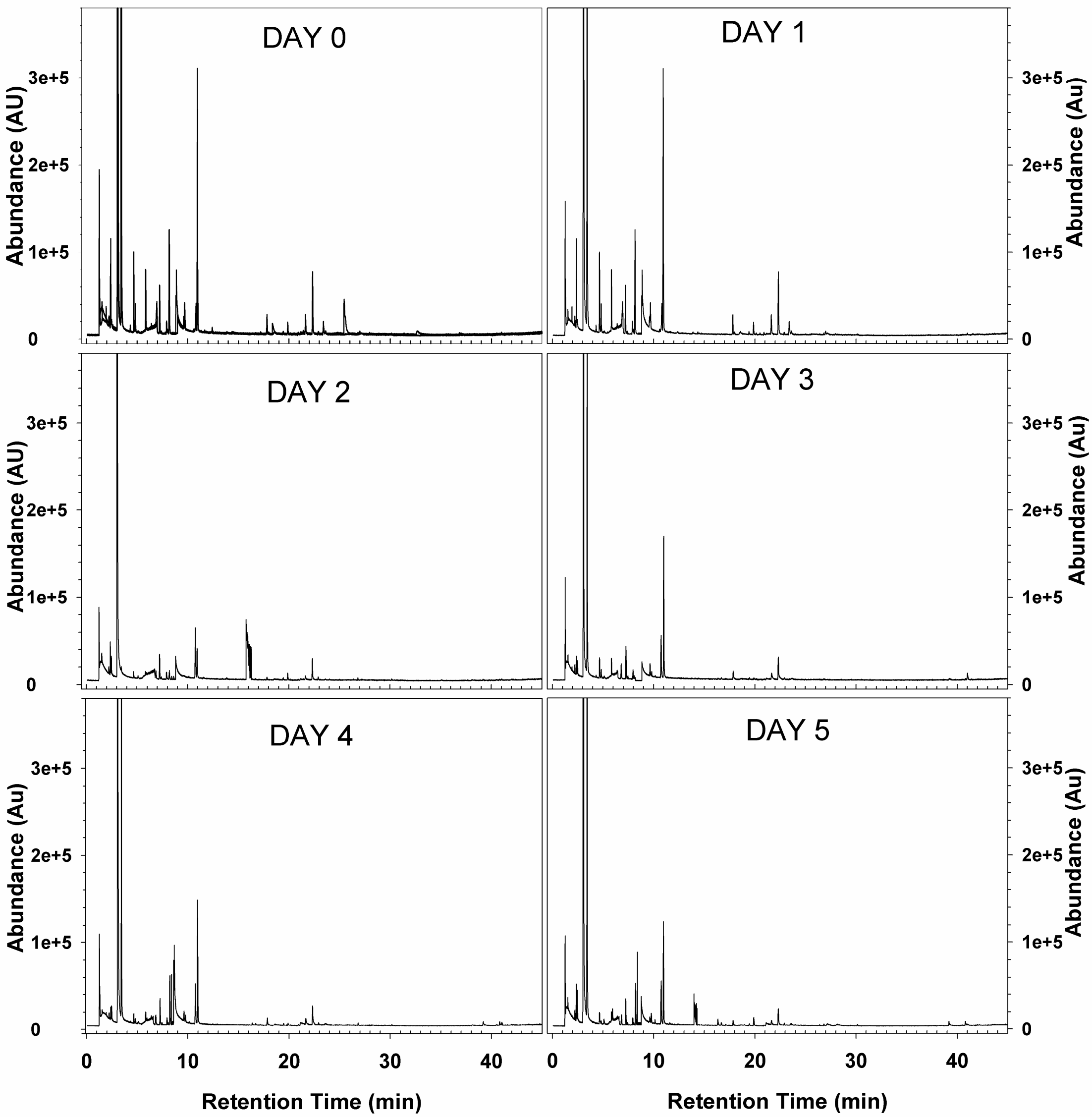

Figure S4. Chromatograms of volatiles from fermented sotol (Dasylirion sp.) must at different days of the process.

Table S1. Identified species in fermented sotol (*Dasylirion* sp) must of significant fermentation applications.

Species	Bioprocess applications
Aspergillus flavus	Production of ascorbic acid [1].
Aspergillus fumigatus	Found in soil and compost piles [2], production of cellulase and xylanase [3].
Aspergillus versicolor	Cocoa beans fermentation, production of xylanase, cellulose, amylase, and pectinase [4].
Bionectria ochroleuca	Production of metabolites of antibacterial, antifungal, and anti-dermatophytic activity [5].
Candida cellae	Ethanol production from banana peel waste [6].
Candida parapsilosis	Sub-Saharan African fermented dairy products [7] and Brazilian Amerindian's cottonseed and rice beverages [8].
Candida tropicalis	Sub-Saharan African fermented cereal-based food and alcoholic beverages [7].
Clavispora lusitaniae	Brazilian Amerindian's cottonseed and rice beverages [8].
Dipodascus australiensis	Mezcal agave based spirit [9].
Galactomyces geotrichum	Mezcal [9] and fermented dairy products [10].
Kodamaea ohmeri	Fermented cocoa beans and pickling [11].
Penicillium chrysogenum	Fermented and cured meat products [12].
Pichia fermentans	Tequila agave-based spirit [13] and wine [14].
Pichia guilliermondii	Tequila agave-based spirit [13].
Pichia kudriavzevii	Tequila agave-based spirit [13] and Chinese liquors [15,16].
Saccharomyces cerevisiae	Tequila, Mezcal, wine, whiskey, and beer [9,13,17].
Wickerhamomyces anomalus	Wine [18].

References of Table S1

1. Banjo, T.; Kareem, S.; Akinduti, P.; Popoola, T.; Akinloye, O. Optimization and production of ascorbic acid by fusant cell of Aspergillus flavus and Aspergillus tamarii. *J. King Saud Univ. - Sci.* **2019**, *31*, 931–936, doi:10.1016/j.jksus.2018.04.032.
2. Willger, S.D.; Grahl, N.; Cramer, R.A., Jr. Aspergillus fumigatus metabolism: Clues to mechanisms of in vivo fungal growth and virulence. *Medical mycology* **2009**, *47 Suppl 1*, S72-S79, doi:10.1080/13693780802455313.
3. Sarkar, N.; Aika, K. Aspergillus fumigatus NITDGPKA3 Provides for Increased Cellulase Production. *Int. J. Chem. Eng.* **2014**, *2014*, 9.
4. de Araújo, J.A.; Ferreira, N.R.; da Silva, S.H.M.; Oliveira, G.; Monteiro, R.C.; Alves, Y.F.M.; Lopes, A.S. Filamentous fungi diversity in the natural fermentation of Amazonian cocoa beans and the microbial enzyme activities. *Ann. Microbiol.* **2019**, *69*, 975–987, doi:10.1007/s13213-019-01488-1.
5. Samaga, P.V.; Rai, V.R.; Rai, K.M.L. Bionectria ochroleuca NOTL33—an endophytic fungus from Nothapodytes foetida producing antimicrobial and free radical scavenging metabolites. *Ann. Microbiol.* **2014**, *64*, 275–285, doi:10.1007/s13213-013-0661-6.
6. Oviedo Zumaqué, L.; Lara Mantilla, C.; Mizger Pantoja, M. Levaduras autóctonas con capacidad fermentativa en la producción de etanol a partir de pulpa de excedentes de plátano Musa (AAB Simmonds) en el departamento de Córdoba, Colombia. *Rev. Colomb. de Biotecnol.* **2009**, *11*, 40–47.
7. Johansen, P.G.; Owusu-Kwarteng, J.; Parkouda, C.; Padonou, S.W.; Jespersen, L. Occurrence and Importance of Yeasts in Indigenous Fermented Food and Beverages Produced in Sub-Saharan Africa. *Front. Microbiol.* **2019**, *10*, doi:10.3389/fmicb.2019.01789.
8. Ramos, C.L.; de Almeida, E.G.; Freire, A.L.; Freitas Schwan, R. Diversity of bacteria and yeast in the naturally fermented cotton seed and rice beverage produced by Brazilian Amerindians. *Food Microbiol.* **2011**, *28*, 1380–1386, doi:10.1016/j.fm.2011.06.012.
9. Jacques-Hernandez, C.; Herrera Perez, O.; Ramírez de León, J. El maguey mezcalero y la agroindustria del mezcal en Tamaulipas. In *En lo ancestral hay futuro: Del tequila, los mezcales y otros agaves*, García Marín, P.C., Larqué Saavedra, A., Eguiarte, L.E., Zizumbo-Villarreal, D., Eds. CICY-CONACYTCONABIO-INE: Tamaulipas, México, 2007; pp. 287–317.
10. Grygier, A.; Myszka, K.; Rudzińska, M. Galactomyces geotrichum - Moulds from dairy products with high biotechnological potential. *Acta Sci. Pol. Technol. Aliment.* **2017**, *16*, 5–16, doi:10.17306/J.AFS.2017.0445.
11. Sharma, S.; Arora, A.; Sharma, P.; Singh, S.; Nain, L.; Paul, D. Notable mixed substrate fermentation by native Kodamaea ohmeri strains isolated from Lagenaria siceraria flowers and ethanol production on paddy straw hydrolysates. *Chem. Cent. J.* **2018**, *12*, 8–8, doi:10.1186/s13065-018-0375-8.
12. Laich, F.; Fierro, F.; Martín, J.F. Production of penicillin by fungi growing on food products: Identification of a complete penicillin gene cluster in Penicillium griseofulvum and a truncated cluster in Penicillium verrucosum. *Appl. Environ. Microbiol.* **2002**, *68*, 1211–1219, doi:10.1128/aem.68.3.1211-1219.2002.
13. Lachance, M.-A. Yeast communities in natural tequila fermentation. *Antonie van Leeuwenhoek* **1995**, *68*, 151–160, doi:10.1007/BF00873100.
14. Clemente-Jimenez, J.M.; Mingorance-Cazorla, L.; Martínez-Rodríguez, S.; Las Heras-Vázquez, F.J.; Rodríguez-Vico, F. Influence of sequential yeast mixtures on wine fermentation. *Int. J. Food Microbiol.* **2005**, *98*, 301–308, doi:10.1016/j.ijfoodmicro.2004.06.007.
15. Yan, S.; Tong, Q.; Guang, J. Yeast dynamics and changes in volatile compounds during the fermentation of the traditional Chinese strong-flavor Daqu. *LWT* **2019**, *106*, doi:10.1016/j.lwt.2019.02.058.
16. Wu, Q.; Xu, Y.; Chen, L. Diversity of yeast species during fermentative process contributing to Chinese Maotai-flavour liquor making. *Lett Appl Microbiol* **2012**, *55*, 301–307, doi:10.1111/j.1472-765X.2012.03294.x.

17. Walker, G.M.; Hill, A.E. Saccharomyces cerevisiae in the Production of Whisky. *Beverages* **2016**, *2*, 38.
18. Padilla, B.; Gil, J.V.; Manzanares, P. Challenges of the Non-Conventional Yeast Wickerhamomyces anomalus in Winemaking. *Fermentation* **2018**, *4*, 68.
